# Enhanced photonics devices based on low temperature plasma-deposited dichlorosilane-based ultra-silicon-rich nitride (Si_8_N)

**DOI:** 10.1038/s41598-022-09227-4

**Published:** 2022-03-28

**Authors:** Doris K. T. Ng, Hongwei Gao, Peng Xing, George F. R. Chen, Xavier X. Chia, Yanmei Cao, Kenny Y. K. Ong, Dawn T. H. Tan

**Affiliations:** 1 Institute of Microelectronics, A*STAR (Agency for Science, Technology and Research), 2 Fusionopolis Way, #08-02, Innovis Tower, Singapore, 138634 Singapore; 2 Photonics Device and Systems Group, Engineering Product Development, Singapore University of Technology and Design (SUTD), 8 Somapah Road, Singapore, 487372 Singapore

**Keywords:** Engineering, Materials science, Optics and photonics

## Abstract

Ultra-silicon-rich nitride with refractive indices ~ 3 possesses high nonlinear refractive index—100× higher than stoichiometric silicon nitride and presents absence of two-photon absorption, making it attractive to be used in nonlinear integrated optics at telecommunications wavelengths. Despite its excellent nonlinear properties, ultra-silicon-rich nitride photonics devices reported so far still have fairly low quality factors of $\sim 6\times {10}^{4}$, which could be mainly attributed by the material absorption bonds. Here, we report low temperature plasma-deposited dichlorosilane-based ultra-silicon-rich nitride (Si_8_N) with lower material absorption bonds, and ~ 2.5× higher quality factors compared to ultra-silicon-rich nitride conventionally prepared with silane-based chemistry. This material is found to be highly rich in silicon with refractive indices of ~ 3.12 at telecommunications wavelengths and atomic concentration ratio Si:N of ~ 8:1. The material morphology, surface roughness and binding energies are also investigated. Optically, the material absorption bonds are quantified and show an overall reduction. Ring resonators fabricated exhibit improved intrinsic quality factors $\sim 1.5\times {10}^{5}$, ~ 2.5× higher compared to conventional silane-based ultra-silicon-rich nitride films. This enhanced quality factor from plasma-deposited dichlorosilane-based ultra-silicon-rich nitride signifies better photonics device performance using these films. A pathway has been opened up for further improved device performance of ultra-silicon-rich nitride photonics devices at material level tailored by choice of different chemistries.

## Introduction

Nonlinear integrated optics promises miniaturization, high field localization and enhanced light-matter interactions^1^. Due to these potential benefits, different types of exotic materials and structures have been explored optically for potential applications in nonlinear optics. Materials that have been investigated include silicon phosphide (SiP)^2^, exfoliated gillulyite flakes^3^, molybdenum diselenide (MoSe_2_)^4^, copper oxide nanoellipsoids^5^, bismuth-doped indium selenide^6^ and many more.

Concurrently, films such as ultra-silicon-rich nitride (USRN)^7–21^ have also been gaining significant interest for nonlinear signal processing due to their complementary metal oxide semiconductor (CMOS) compatibility and excellent nonlinear device performance at telecommunications wavelengths. Compared to silicon (Si), USRN has proven absence of 2-photon absorption and free carrier losses^9^, which are common in Si^22,23^. Compared to stoichiometric silicon nitride (Si_3_N_4_)^24,25^, the nonlinear refractive index of USRN is reported^9,11^ to be 100× larger in magnitude. Notably, USRN films have demonstrated high Kerr nonlinearities^11^ of 2.8 × 10^–17^ m^2^/W^7^, enabling nonlinear photonic devices for high optical parametric gain of 42.5 dB^7^, high spectro-temporal compression^8^, wideband spectral broadening^15^, optical parametric Bragg amplification^16^ and observations of Bragg soliton pheonomena^14,26,27^. Using lower temperatures < 400 °C to prepare the USRN films by plasma-deposited methods has also been attractive due to the flexibility to fabricate USRN photonics devices on CMOS electronics layers. This provides the motivation to explore USRN films prepared at low temperatures < 400 °C. So far, USRN films, as well as other formulations of silicon-rich nitride reported for optical signal processing have been prepared using silane (SiH_4_)-based plasma deposition at temperatures of ~ 250–350 °C^18–21,28^. Preparing plasma-deposited USRN films using dichlorosilane (DCS, SiH_2_Cl_2_)-based chemistry at low temperatures of ~ 300 °C has, to date, been relatively unexplored. DCS-based chemistry is very commonly used in high temperature (~ 800 °C) low pressure chemical vapor deposition (LPCVD)^29–32^ processes to prepare silicon nitride (SiN) films but the high thermal budget will hinder monolithic integration with CMOS electronics layers.

To the best of our knowledge, this is the first demonstration of Si_8_N USRN photonics device prepared using DCS-based plasma process at a low temperature ~ 300 °C. Using DCS-based chemistry, we prepare plasma-deposited USRN films and conduct in-depth investigations on them. In addition to characterizing the films’ material and optical properties, their performance as photonics devices are also measured. This DCS-based USRN film is amorphous with a surface roughness root-mean-square (rms) of ~ 0.4 nm. Characterization of the films’ atomic composition indicates a high Si concentration with Si:N ratio ~ 8:1, indicating Si_8_N. At 1550 nm wavelength, the refractive index is ~ 3.12 due to its ultra-silicon-rich content, with negligible extinction coefficient. Further studies and quantification of the material absorption bonds reveal overall less absorption bonds compared to conventional SiH_4_-based USRN film^7–17^. Although the overall absorption bonds drop by ~ 12%, we note a drop of ~ 40% in Si–H bonds in DCS-based USRN film. Waveguide devices with ring resonators fabricated using these DCS-based USRN films measure intrinsic quality factors exceeding 10^5^, ~ 2.5× higher than that of SiH_4_-based USRN films^9^ used in nonlinear photonics devices. The ~ 40% reduction in Si–H bonds contributes to a certain extent, the 2.5× increase in quality factor. Reduced material absorption presents opportunities for CMOS-compatible USRN devices with improved device performance, important for nonlinear optics applications including parametric wavelength conversion, frequency comb generation and temporal compression dynamics.

## Results and discussion

### Characterization of DCS-based USRN film

**Figure 1:**
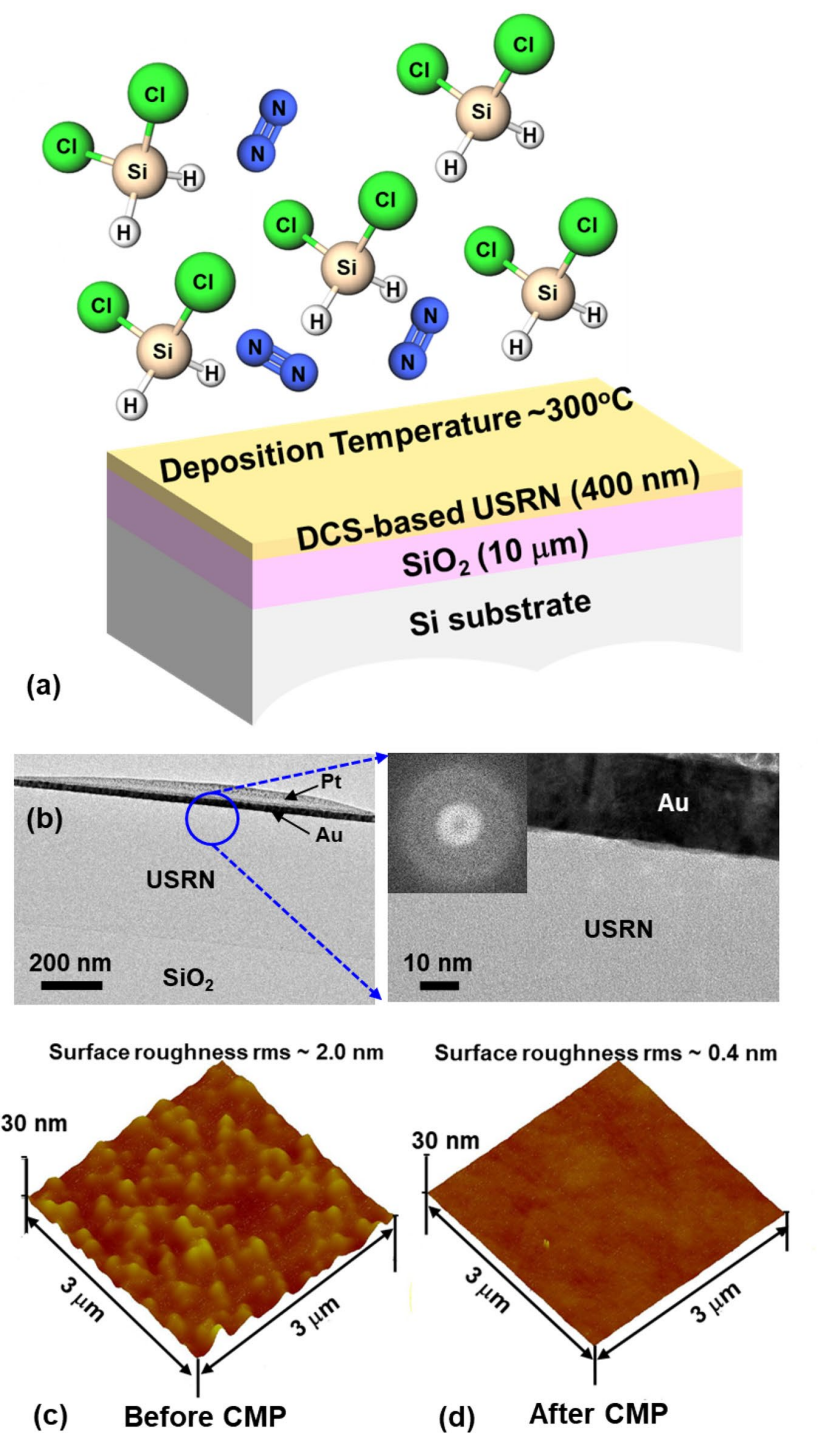
(**a**) Schematic drawing of the plasma-deposited DCS-based USRN film of ~ 400 nm thickness deposited at low temperature ~ 300 °C with DCS and N_2_ gas precursors. (**b**) Cross-sectional TEM image of the deposited DCS-based USRN film showing Au and Pt layers on the top, deposited due to TEM sample preparation by FIB-cut. Below the USRN film is a thermal SiO_2_ layer. High resolution TEM image is taken at the region marked by the blue circle. This close-up image did not reveal any lattice structure, indicating that the film is amorphous. An FFT analysis (inset) on the DCS-based USRN film further confirm that the film is amorphous as no distinct bright spot is observed. AFM measuring the surface roughness of DCS-based USRN film (**c**) before and (**d**) after CMP. Surface roughness rms is reduced from ~ 2.0 to ~ 0.4 nm by CMP.

A series of material characterizations is performed on the DCS-based USRN film to determine the characteristics of this film. Figure 1a shows a schematic drawing of the designed USRN film, plasma-deposited using DCS-based chemistry at a low temperature ~ 300 °C with DCS and nitrogen (N_2_) gas precursors on top of a 10 μm thick thermally grown silicon dioxide (SiO_2_) with Si as the substrate. Figure 1b shows the cross-sectional transmission electron microscopy (TEM) images of DCS-based USRN film on thermal SiO_2_ layer. The TEM image shows DCS-based USRN film of thickness of ~ 400 nm. The platinum (Pt) and gold (Au) layers on top of USRN film are deposited due to TEM sample preparation as focused ion beam (FIB) is used to cut across the film for cross-sectional TEM imaging. A high resolution cross-sectional TEM image is taken near the top surface of the film, indicated by the blue circle. With a scale bar of 10 nm, we observe that the DCS-based USRN film seems amorphous. This is further confirmed using fast fourier transform (FFT) analysis as depicted in the inset. The FFT analysis shows no distinct bright spots, indicating that the film is amorphous. Atomic force microscopy (AFM) is used to examine the surface roughness of DCS-based USRN film. Figure 1c,d show AFM images of DCS-based USRN film before and after chemical–mechanical polishing (CMP) process respectively. The scan area used is 3 μm × 3 μm. DCS-based USRN film surface roughness rms measures ~ 2.0 nm before CMP process (Fig. 1c). With CMP process done after the film is deposited, the surface is smoothened to ~ 0.4 nm rms, which is close to substrate roughness rms of ~ 0.2 nm. Surface roughness is important for photonics devices as rougher surfaces will induce scattering losses as light propagates through the waveguides.

**Figure 2:**
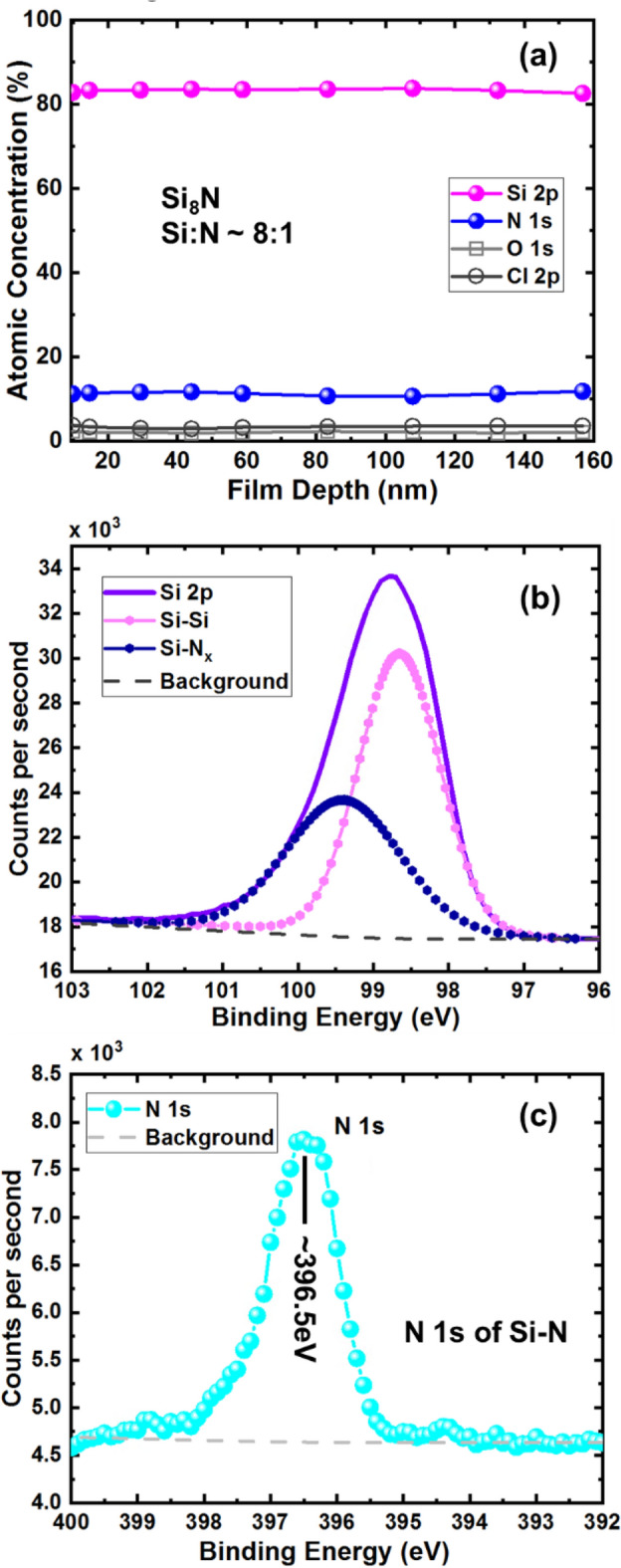
(**a**) XPS depth profile showing atomic concentration in the DCS-based USRN films from depth at surface of ~ 10 to ~ 160 nm into the film. Si 2p shows atomic concentration ~ 83% while N 1s shows atomic concentration ~ 11%, indicating Si:N ratio ~ 8:1. We infer that the film composition is Si_8_N. There are minimal O 1s (~ 2%) and Cl 2p (~ 3%) in the film. (**b**) Narrow scan XPS spectrum from 103 to 96 eV binding energies revealing Si 2p peak. This spectrum is further deconvoluted to identify Si–Si peak and Si–N peak. XPS background has been taken into account of. (**c**) Narrow scan XPS spectrum from 400 to 392 eV binding energies showing N 1s peak at ~ 396.5 eV, typical binding energy of N in Si–N films, indicating N elements bonded to Si elements in DCS-based USRN (Si_8_N) film. XPS background has been taken into account of.

Figure 2a shows the X-ray photoelectron spectroscopy (XPS) depth profile of DCS-based USRN films. The atomic concentration in the film is measured from a depth of ~ 10 to ~ 160 nm. The depth profile reveals presence of Si 2p, N 1s and also small amount of O 1s and Cl 2p. The atomic concentration of Si 2p is ~ 83% while N 1s shows atomic concentration ~ 11%. The ratio of Si:N could be read as ~ 8:1. Using this ratio and having DCS and N_2_ gas as the reacting gases, we derive the following chemical reaction for the deposited USRN film:

1 $${\text{16SiH}}_{{2}} {\text{Cl}}_{{2}} + {\text{ N}}_{{2}} \to {\text{ 2Si}}_{{8}} {\text{N }} + {\text{ 32HCl}}.$$

SiH_2_Cl_2_ (DCS) and N_2_ reacts to form Si_8_N (USRN) and hydrochloric (HCl). O 1s and Cl 2p are also detected at small quantities with atomic concentration of ~ 2% and ~ 3% respectively. The presence of O 1s could be caused by chamber condition and handling of wafer while the presence of Cl is most likely from the DCS gas used which contains Cl content. Figure 2b shows an XPS narrow scan of Si 2p from binding energy range of 103–96 eV. This Si 2p spectrum peaks at ~ 98.8 eV binding energy. The spectrum is further deconvoluted into 2 curves with peak at ~ 98.7 eV identified as Si–Si and peak at ~ 99.4 eV identified as Si–N_x_^7^. The higher counts of Si–Si compared to Si–N_x_ is further indication of the Si-rich nature of the film.

Figure 2c shows XPS narrow scan of N 1s at binding energy at ~ 396.5 eV, typical of N 1s peak of Si–N^33^, indicating that N element is bonded with Si element in the USRN (Si_8_N) film. We note that the N 1s counts of DCS-based USRN obtained in Fig. 2c is ~ 7.82 × 10^3^ counts per second, lower than N 1s counts of SiH_4_-based USRN film^7^ which reports ~ 12.8 × 10^3^ counts per second. This could mean DCS-based USRN is less N-rich than SiH_4_-based USRN film, due to the lower N counts in the DCS-based USRN film. Taking XPS background signal into consideration, we try to estimate the ratio of N 1s for DCS-based USRN with that of SiH_4_-based USRN film reported in literature^7^. We estimate a ratio ~ 0.66 (< 1) for N 1s (DCS-based USRN: SiH_4_-based USRN), which seems to indicate that DCS-based USRN contains less N than SiH_4_-based USRN.

**Figure 3:**
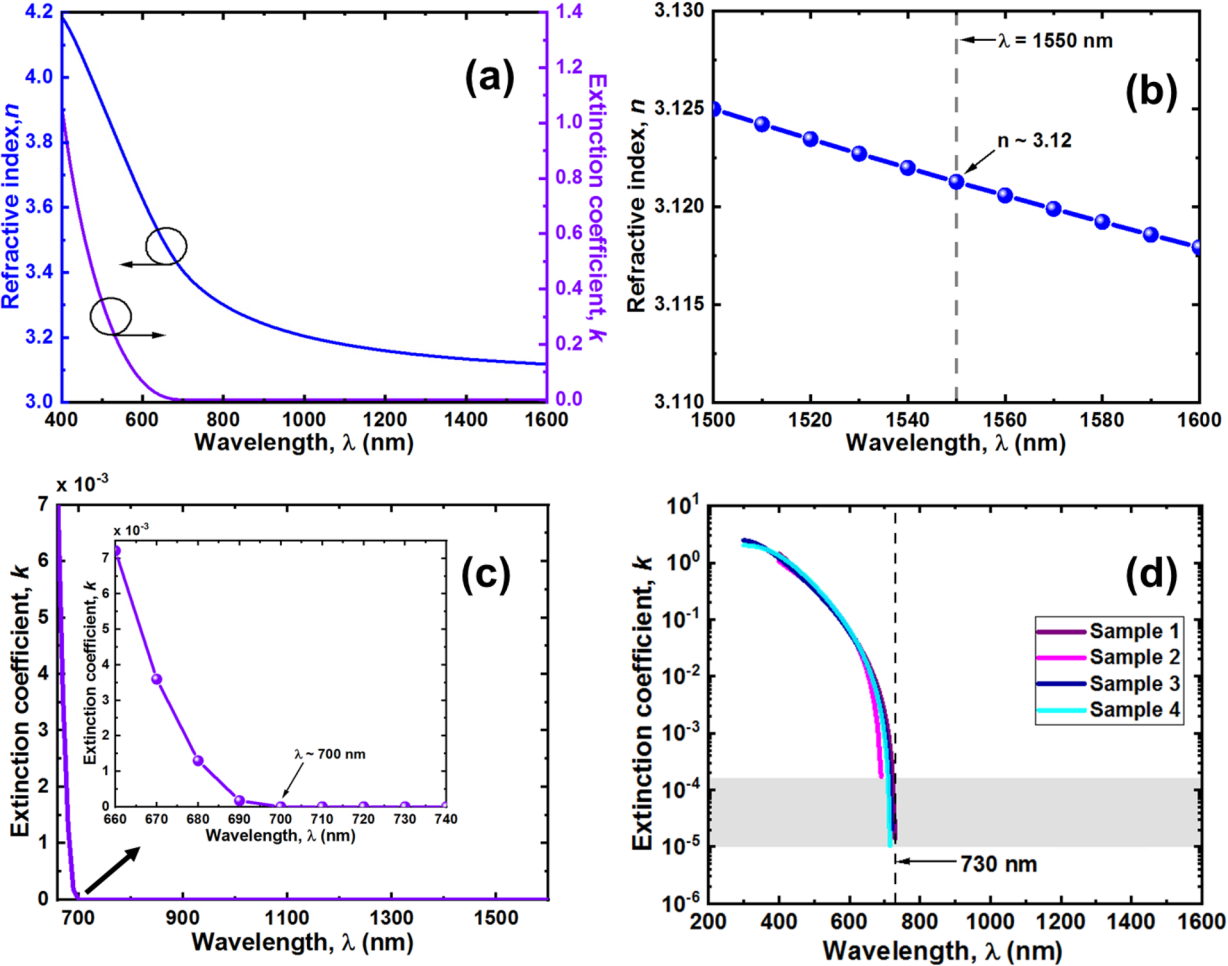
(**a**) Dispersion spectra of DCS-based USRN films characterized using a variable-angle spectroscopic ellipsometry over wavelength range from 400 to 1600 nm. Film refractive index, *n* is shown by the blue line (corresponds to the left vertical axis) and extinction coefficient, *k* is shown by the purple line (corresponds to the right vertical axis). (**b**) Magnified wavelength range from 1500 to 1600 nm (in steps of 10 nm) showing refractive index, *n* of DCS-based USRN film ~ 3.12 at 1550 nm wavelength. (**c**) Extinction coefficient, *k* of DCS-based USRN film from ~ 660 to 1600 nm wavelength range. The graph shows *k* reducing to 0 in the 700 nm wavelength region. Inset shows zoom-in around 700 nm wavelength region and reveal *k* ~ 0 (negligible) at λ ~ 700 nm. (**d**) Extinction coefficient, *k* of 4 DCS-based USRN film samples with *k* plotted in logarithm scale. The lowest k in this case is ~ 10^–5^ which occurs at ~ 730 nm wavelength.

Figure 3a shows the dispersion spectra of DCS-based USRN film measured across wavelength range from 400 to 1600 nm. The left vertical axis indicates the film’s refractive index, *n* denoted by the blue plot while the right vertical axis indicates the film’s extinction coefficient, *k* denoted by the purple plot. The refractive index plot is further zoom-in in Fig. 3b plotting over the wavelength range from 1500 to 1600 nm in steps of 10 nm. At our wavelength of interest 1550 nm, refractive index, n ~ 3.12 which is close to that of amorphous Si (*n* ~ 3.4)^34^, exhibiting the ultra-silicon-rich nature of the film. Figure 3c shows the plot of DCS-based USRN film extinction coefficient, *k* in the wavelength region from 660 to 1600 nm where *k* starts to decrease to 0 and remain at 0 till λ = 1600 nm. Inset shows the zoom-in plot at around 700 nm wavelength region. We note that *k* decreases to 0 starting from wavelength ~ 700 to 1600 nm, showing that the film absorption is minimal or negligible starting from wavelength of ~ 700 nm. To look more precisely into *k*, we measure *k* across wavelengths for 4 DCS-based USRN films and plot *k* in logarithmic against wavelength. Figure 3d shows graph of *k* across wavelengths ~ 300–1600 nm for 4 DCS-based USRN films. We note that *k* decreases to the range ~ 10^–4^–10^–5^ at wavelengths ~ 690–730 nm respectively. Beyond that, *k* cannot be detected by the ellipsometer. This film will thus exhibits higher absorption loss at wavelengths < 700 nm, hence not suitable for photonics devices working at visible wavelengths < 700 nm.

**Figure 4:**
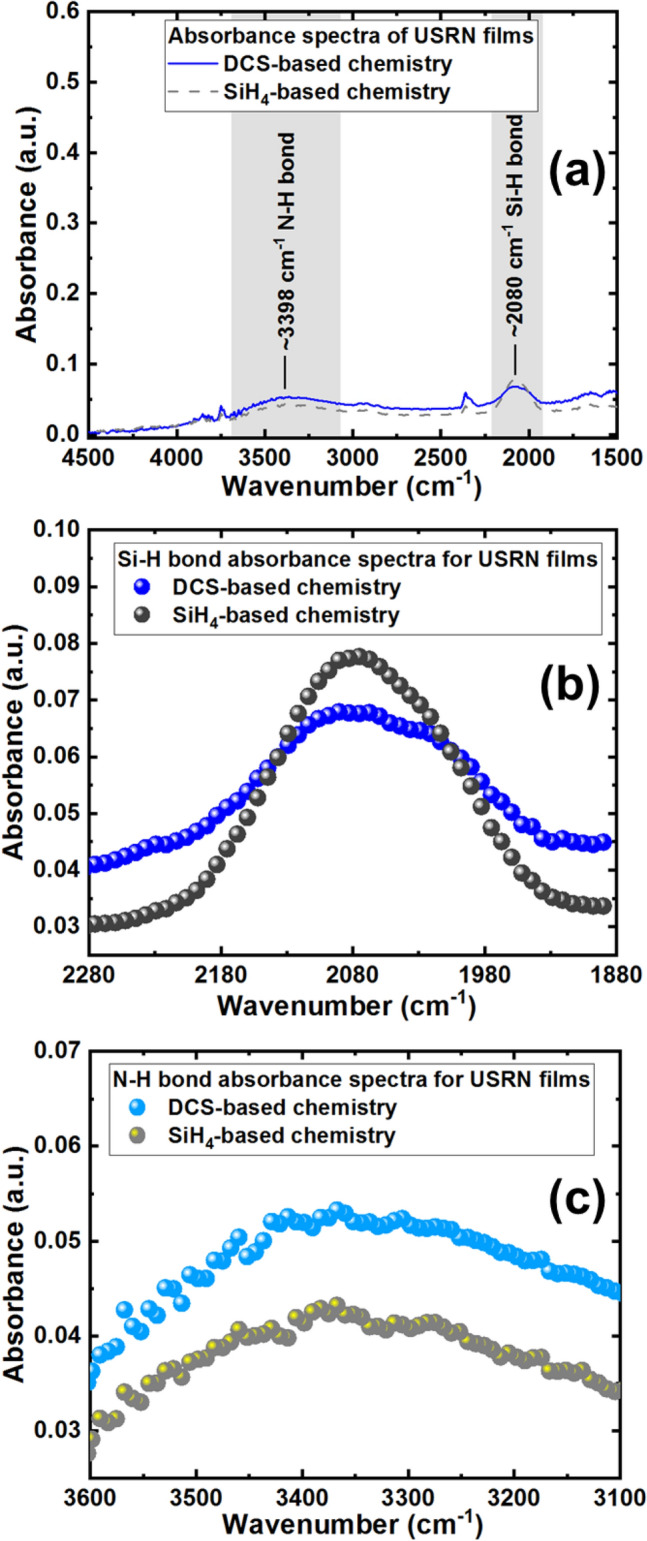
FTIR measurements showing (**a**) the absorbance spectra of USRN films covering from wavenumber 1500 to 4500 cm^−1^. The USRN films are prepared using DCS-based chemistry (absorption spectrum in blue line) and SiH_4_-based chemistry (absorption spectrum in dashed grey line). Si–H bond is identified at peak ~ 2080 cm^−1^, spanning from ~ 1880 to 2280 cm^−1^ (marked by the grey region) and N–H bond is identified at peak ~ 3398 cm^−1^, spanning from ~ 3100 to 3600 cm^−1^ (marked by the 2nd grey region), (**b**) Si–H bond absorbance spectra from wavenumber range 1880 to 2280 cm^−1^ for the 2 USRN films prepared using different gas chemistries and (**c**) N–H bond absorbance spectra from wavenumber range 3100 to 3600 cm^−1^ for the 2 USRN films prepared using different gas chemistries.

Figure 4a depicts the fourier transform infrared (FTIR) spectra of USRN films measured using attenuated total reflection (ATR) technique. Absorbance is calculated and plotted using the following equation:

2 $$Abs=2-{log}_{10}\left(T\%\right),$$

where *Abs* is absorbance and *T%* is the transmission in percentage obtained directly from FTIR ATR measurement. A comparison is done between DCS-based USRN film (Si_8_N) and SiH_4_-based USRN film prepared based on Ref.^28^. The FTIR spectra reveal presence of N–H and Si–H absorption bonds in both films. Regions where Si–H and N–H bonds could occur are indicated by the grey regions—wavenumber range from ~ 1880 to 2280 cm^−1^ for Si–H bond region and wavenumber range from ~ 3100 to 3600 cm^−1^ for N–H bond region. Si–H bond peak is identified at wavenumber ~ 2080 cm^−1^ while N–H bond peak is identified at wavenumber ~ 3398 cm^−1^, in agreement with what was previously reported for ultra-silicon-rich nitride films^35^. The more prominent Si–H peak compared to N–H peak could be due to the ultra-silicon-rich content in the film.

Figure 4b shows a zoom-in FTIR plot in the Si–H bond region from wavenumber range 1880–2280 cm^−1^ for both SiH_4_-based and DCS-based USRN films while Fig. 4c shows the FTIR plot for both films in the N–H bond region from wavenumber range 3100–3600 cm^−1^. We note that Si–H absorption seems higher in SiH_4_-based USRN films compared to DCS-based USRN films while the N–H absorption seems comparable in both USRN films. To study this further, we try to quantify the Si–H bonds and N–H bonds using Lanford and Rand’s technique^36^, based on the following equations^36–38^:

3 $$\left[\text{Si}{-}\text{H}\right]=\frac{1}{2.303\times {\sigma }_{\text{Si}{-}\text{H}}}\times {\int }_{band}\alpha \left(\omega \right)d\omega ,$$

4 $$\left[\text{N}{-}\text{H}\right]=\frac{1}{2.303\times {\sigma }_{\text{N}{-}\text{H}}}\times {\int }_{band}\alpha \left(\omega \right)d\omega ,$$

where ${\sigma }_{\text{Si}{-}\text{H}}=7.4\times {10}^{-18} \,{\text{cm}}^{2}$ and ${\sigma }_{\text{N}{-}\text{H}}=5.3\times {10}^{-18} \,{\text{cm}}^{2}$ are the absorption cross-sections of Si–H and N–H respectively. ${\int }_{band}\alpha (\omega )d\omega$ is the normalized absorption area of the band. $\alpha =(2.303/t)A$ where $\alpha$ is the absorption coefficient, $A$ is the absorbance and $t$ is the film thickness which is 400 nm.

Table 1 shows a summary of the Si–H and N–H bond calculated from the FTIR absorbance spectra of DCS-based USRN film (in this work) and SiH_4_-based USRN films (prepared based on Ref.^28^). We note that in both films, the Si–H and N–H bond concentrations are in the range of 10^22^ cm^−3^. The Si–H bond concentration calculated from DCS-based USRN film shows lower Si–H bonds of $\sim 1.47\times {10}^{22 }{\text{ cm}}^{-3}$ compared to that of SiH_4_-based Si–H bonds of $\sim 2.45\times {10}^{22} \,{\text{cm}}^{-3}$, in agreement with FTIR plots shown in Fig. 4b that shows smaller Si–H bond plots for DCS-based USRN film. The N–H bond concentration for DCS-based USRN film however is calculated to be slightly higher ($\sim 2.80\times {10}^{22}{\text{ cm}}^{-3}$) than SiH_4_-based USRN film ($\sim 2.42\times {10}^{22}{ \text{cm}}^{-3}$). A further calculation of ratio [N–H]/[Si–H] returns ~ 1.91 for DCS-based USRN film and ~ 0.99 for SiH_4_-based USRN film, indicating higher N–H than Si–H bond concentrations in DCS-based USRN film. This is in contrary to what was reported in literature^18,39,40^ where one would expect to see more Si–H bonds compared to N–H bonds in Si-rich nitride. The reason for the lower Si–H bonds in this work is most likely due to the gas chemistry used to form USRN films. The Si-rich nitride films previously reported^18,39,40^ were deposited using SiH_4_-based gas chemistries while here, we are using DCS-based gas chemistry to form the USRN film. The differences in the gases used could have changed the bond concentrations, and in this case we note that DCS-based gas chemistry seems to play a major role in reducing Si–H bonds. There is less hydrogen (H) content in DCS gas compared to SiH_4_ gas, which could result in less Si–H bonds. Table 1 shows a ~ 40% drop in Si–H bonds and ~ 15% increase in the N–H bonds in the DCS-based USRN film when compared to SiH_4_-based USRN film. A total of Si–H and N–H bond concentrations is then calculated for each film to account for the overall material absorption loss due to these bonds. A bond concentration of $\sim 4.27\times {10}^{22}{ \text{cm}}^{-3}$ in the DCS-based USRN film and $\sim 4.87\times {10}^{22} \,{\text{cm}}^{-3}$ in the SiH_4_-based USRN film is calculated, representing a drop of ~ 12% in overall absorption bonds. Although we are seeing an overall drop of a mere ~ 12% in the absorption bonds, the significant ~ 40% drop in the Si–H bonds is considerable and would have impact on improved losses in the photonic devices.

**Table 1:** Summary of Si–H and N–H bond concentrations from DCS-based and SiH_4_-based USRN films, showing the percentage change in bond concentrations for DCS-based USRN film when compared to SiH_4_-based USRN film.

Film description	Bond concentration (cm^−3^)			[N–H]/[Si–H]
	Si–H	N–H	Si–H and N–H	
DCS-based USRN film (this work)	1.47 × 10^22^	2.80 × 10^22^	4.27 × 10^22^	1.91
SiH_4_-based USRN film (prepared based on Ref.^28^)	2.45 × 10^22^	2.42 × 10^22^	4.87 × 10^22^	0.99
% Change in bond concentration	40% (decrease)	15% (increase)	12% (decrease)	

The bond concentration ratios [N–H]/[Si–H] are also calculated for both films.

Si–H and N–H bonds are undesirable material absorption bonds in photonic waveguide-based devices working at wavelength region ~ 1550 nm as they will cause light propagation loss due to material absorption loss in the waveguides and affect quality factors in waveguide resonator devices. Looking into the potential impact of these absorption bonds on the performance of photonic devices, we note that from the FTIR data in Fig. 4, Si–H fundamental absorption peak is at wavenumber ~ 2080 cm^−1^, which corresponds to wavelength ~ 4807.69 nm. We would expect a second overtone peak at ~ 1602.56 nm wavelength. N–H fundamental absorption peak measured is at wavenumber ~ 3398 cm^−1^ which corresponds to wavelength ~ 2942.91 nm, with first overtone peak at ~ 1471.46 nm wavelength. Photonic devices measured in the wavelength range that overlap with these Si–H second overtone peak and N–H first overtone peak will be affected by these absorption peaks, with some losses caused by the material absorption.

This series of material characterizations help to validate suitability of DCS-based USRN for photonics applications at telecommunications wavelength. Results have revealed that these DCS-based USRN films are amorphous and have a smooth surface roughness rms ~ 0.4 nm, useful information for consideration during device design. The high Si content desired for the material to be ultra-silicon-rich is confirmed by XPS which gives a high Si:N ratio of 8:1, and the material’s ultra-high refractive index. As Si content is very high in the film, XPS has also help to confirm presence of N in the film, so as not to mistake these films as amorphous Si. Looking into the transparency of these films, an ellipsometry scan has revealed that maximum transmittance starts at ~ 690–730 nm wavelength range, by the starting wavelength point where negligible absorption coefficient is detected, indicating lower propagation losses towards telecommunications wavelengths. Further optical characterization via FTIR reveals the presence of Si–H and N–H absorption bonds in the material. Upon quantification of these bonds, we note that DCS-based USRN films have overall less absorption bonds and quite a significant drop (~ 40%) in Si–H absorption bonds compared to SiH_4_-based USRN films. We proceed to fabricate photonics devices using DCS-based USRN film and characterize them at device level.

### Device characterization

**Figure 5:**
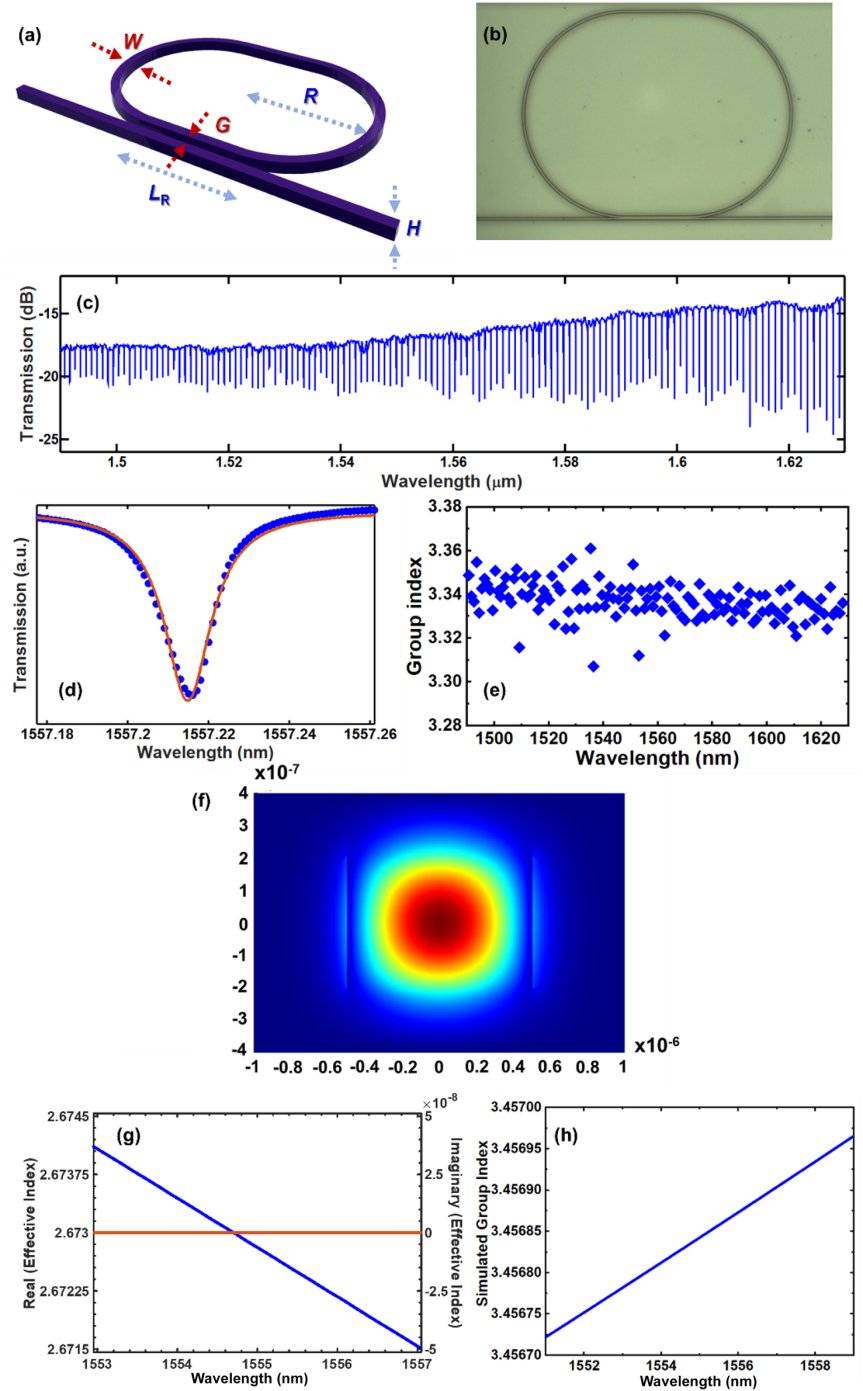
(**a**) Schematic and (**b**) optical micrograph of the racetrack ring resonators. (**c**) Measured transmission spectrum of ring resonator with *H* = 400 nm, *W* = 1000 nm, *R* = 100 μm, *G* = 200 nm, racetrack length, *L*_R_ = 30 μm. (**d**) Lorentzian fitting of a single resonance. (**e**) Group index spectrum of the resonator. (**f**) Simulated mode profile for Si_8_N waveguide at 1557 nm. (**g**) Simulated real (blue) and imaginary (orange) effective refractive index for Si_8_N waveguide (*W* = 1000 nm, *H* = 400 nm). (**h**) Simulated group index for Si_8_N waveguide (*W* = 1000 nm, *H* = 400 nm).

The fabricated devices are characterized using a tunable continuous-wave laser spanning from 1490 to 1630 nm. The polarization of the light is adjusted to select transverse-electric polarization before coupling into the devices terminated with inverse tapers. The schematic of the racetrack ring resonators is shown in Fig. 5a while Fig. 5b shows an optical micrograph of the fabricated device. A Lorentzian fit is applied to the resonances of each characterized ring resonator using the equation^41,42^:

5 $${Q}_{L}=\frac{\lambda }{\Delta \lambda },$$

where Δ*λ* is the full width at half maximum. The intrinsic quality factor of the resonators may then be extracted using:

6 $${Q}_{i}=\frac{2{Q}_{L}}{1\pm \sqrt{T}},$$

where *T* is the transmission at resonance, $\pm$ refers to the coupling state of the resonator. Our resonators are under coupled, hence we use “–” in (6). The waveguide propagation loss associated with the resonators, *α* may be calculated using^43^:

7 $$\alpha =\frac{2\pi {n}_{g}}{{Q}_{i}\lambda }.$$

The group index *n*_g_ is a function of wavelength, and may be obtained from the resonator transmission spectrum using:

8 $${n}_{g}=\frac{{\lambda }^{2}}{2\left(\pi R+{L}_{R}\right) \times FSR},$$

where *R* and FSR are the ring radius free spectral range respectively. The measured transmission spectrum of devices with waveguide height, *H* = 400 nm, width, *W* = 1000 nm, radius, *R* = 100 μm, gap, *G* = 200 nm and racetrack length, *L*_R_ = 30 μm is shown in Fig. 5c, the loaded quality factor is extracted as $\sim 1.1\times {10}^{5}$, as shown in Fig. 5d. The corresponding intrinsic quality factor of $\sim 1.37\times {10}^{5}$ and ~ 2.4 dB/cm waveguide propagation loss. The group index is calculated and plotted in Fig. 5e. To verify the measured result, the waveguide mode profile (Fig. 5f), effective refractive index (Fig. 5g), and group index (Fig. 5h) of the structure are calculated and compared with our experimental results. As seen from Fig. 5h, the group index calculated is close to the extracted value based on the measured transmission spectrum.

**Figure 6:**
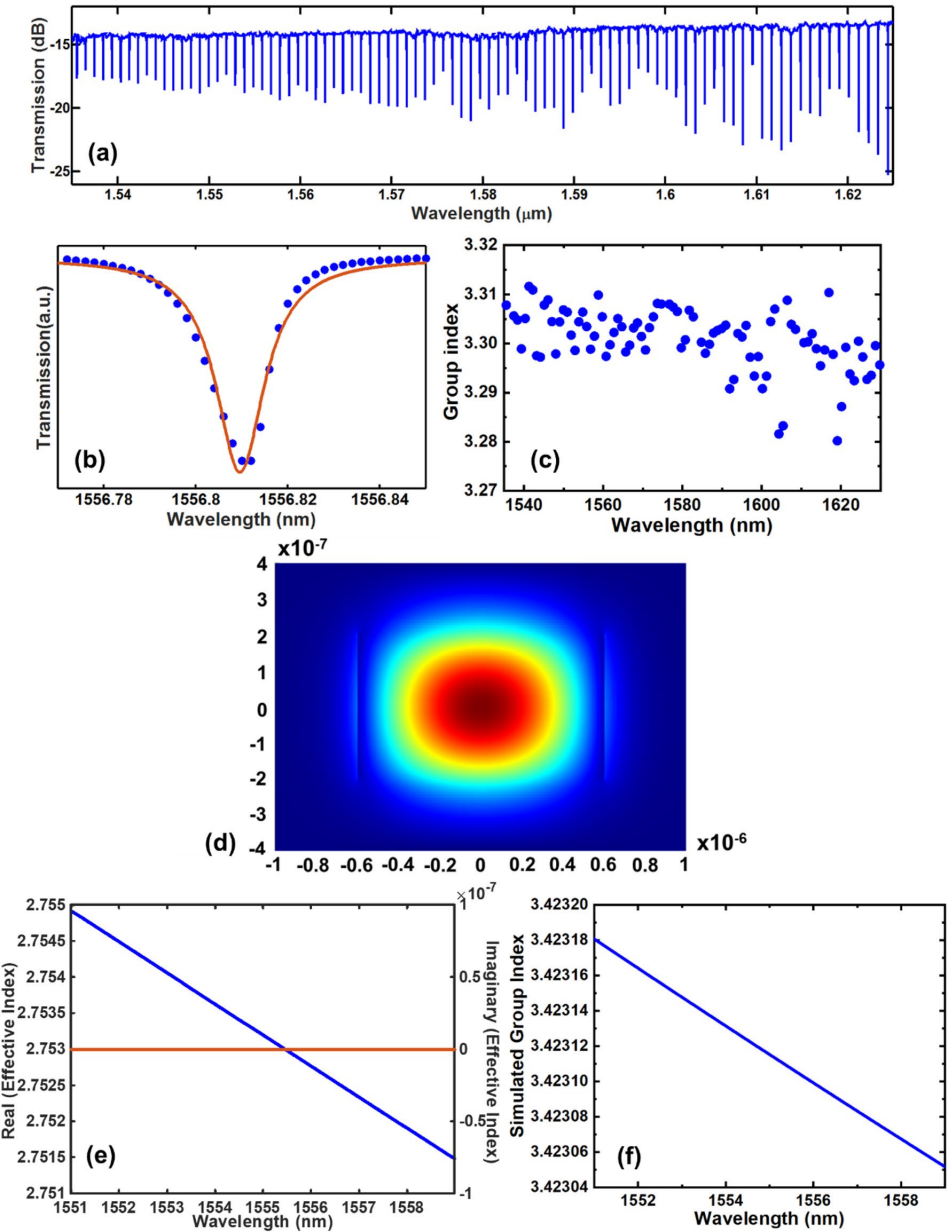
(**a**) Measured transmission spectrum of ring resonator with *H* = 400 nm, *W* = 1200 nm, *R* = 100 μm, *G* = 200 nm and *L*_R_ = 60 μm. (**b**) Lorentzian fitting of a single resonance. (**c**) Group index as a function of wavelength. (**d**) Simulated mode profile for Si_8_N waveguide at 1557 nm. (**e**) Simulated real (blue) and imaginary (orange) effective refractive index for Si_8_N waveguide (*W* = 1200 nm, *H* = 400 nm), (**f**) simulated group index for Si_8_N waveguide (*W* = 1200 nm, *H* = 400 nm).

The measured transmission spectrum of devices with waveguide width of 1200 nm is further shown in Fig. 6a. The loaded quality factor is extracted as $\sim 1.22\times {10}^{5}$, as shown in Fig. 6b. The corresponding intrinsic quality factor and propagation loss are $\sim 1.53\times {10}^{5}$ and ~ 2.2 dB/cm respectively. The group index is further calculated and plotted in Fig. 6c. Numerical calculation of the mode profile, effective refractive index and group index is shown in Fig. 6d–f respectively. Figure 6f shows that the group index is close to the extracted value from the measured transmission spectrum (Fig. 6c).

Table 2 shows comparison of the quality factors (both intrinsic and loaded) extracted from DCS-based USRN ring resonators (Figs. 5, 6) and SiH_4_-based USRN ring resonators reported in Ref.^9^. We note that the DCS-based USRN film enables ring resonators with intrinsic quality factors ~ 2.5× higher than those fabricated with SiH_4_-based USRN films. Ring resonators based on SiH_4_-based USRN films were previously reported^9^ to have quality factors of $\sim 6.0\times {10}^{4}$ (intrinsic) and $\sim 4.5\times {10}^{4}$ (loaded). The devices fabricated using DCS-based USRN films are measured in the wavelength range between 1490 and 1630 nm. From the FTIR measurement (Fig. 4), Si–H second overtone absorption peak occurs at a wavelength of ~ 1602.56 nm and the N–H first overtone absorption peak occurs at a wavelength of ~ 1471.46 nm. The device measurement wavelength range covers the Si–H second overtone absorption peak but avoids the first N–H overtone absorption peak. Hence, in the wavelength range where the devices are measured, Si–H second overtone absorption peak will have more impact on the device performance compared to N–H first overtone absorption peak. In addition, Si–H second overtone absorption peak is closer to 1550 nm wavelength than N–H first overtone absorption peak. It is also worthwhile to note that although N–H first overtone absorption peak is avoided, the tail of the N–H absorption spectrum did overlap into this wavelength range and to some extent, will affect the device performance. The drop in Si–H bond concentration of ~ 40% for DCS-based USRN film compared to SiH_4_-based USRN film could have played a major role in the quality factor increase for DCS-based USRN devices. At device fabrication level, while DCS-based USRN film’s surface roughness of ~ 0.4 nm is low, further loss reductions through process optimization to further reduce etched sidewall roughness and surface roughness is possible. Loss mechanisms in integrated photonic devices is multi-faceted, with small improvements requiring extensive process tweaks. However, their salience in device performance, particularly in supporting resonant enhanced mechanisms stems from scaling laws with the resonator quality factor. For example, as pertains to optical sensing applications, the effective optical path length of a resonator scales linearly with the quality factor^44^. In nonlinear applications such as parametric wavelength conversion in a resonator^45,46^, there exists a superlinear scaling with the resonator quality factor, where the conversion efficiency scales with the square of the resonator finesse^47^. Quantum optical applications such as resonant-enhanced photon pair generation also benefit significantly from the resonator finesse^48,49^. In this case, the finesse calculated for the DCS-based USRN ring resonators are shown in Table 2. Given the large linear refractive index of 3.12 in DCS-based USRN, ring resonators with smaller radius closer to 50 μm could also possess negligible bending losses. In the future, more compact resonators could be developed to widen the resonator FSR while maintaining or improving the quality factor, and in so forth achieve resonators of higher finesse using DCS-based USRN. Consequently, multiplicative improvements to application specific photonic device performance may be derived from loss reduction efforts.

**Table 2:** Comparison of quality factors extracted from DCS-based USRN resonators (in this work) with SiH_4_-based USRN resonators (Ref.^9^).

Quality factor	DCS-based USRN (Fig. 5)	DCS-based USRN (Fig. 6)	SiH_4_-based USRN^9^
Intrinsic	$\sim 1.37\times {10}^{5}$	$\sim 1.53\times {10}^{5}$	$\sim 6.0\times {10}^{4}$
Loaded	$\sim 1.1\times {10}^{5}$	$\sim 1.22\times {10}^{5}$	$\sim 4.5\times {10}^{4}$
Finesse	~ 75	~ 77	

The finesse of the DCS-based USRN devices are also calculated here.

## Conclusion

We have prepared CMOS-compatible USRN films using DCS-based chemistry, plasma deposited at a low temperature of ~ 300 °C. These DCS-based films prepared are amorphous as characterized by TEM. Surface roughness rms of the film measures ~ 0.4 nm. An XPS analysis on the films’ composition indicates that this film is Si_8_N, as it reveals high Si:N ratio with ~ 80% of Si and ~ 10% of N with deconvolution of the Si 2p curve showing Si–Si bonds and Si–N_x_ bonds. Optically, these DCS-based USRN films exhibit a refractive index of ~ 3.12 at 1550 nm wavelength, indicating the ultra-silicon-rich content in the films. The films’ extinction coefficient is negligible within the measured wavelength range of ~ 700 nm to 1600 nm. Although we detect the presence of N–H and Si–H absorption bonds with FTIR spectroscopy, overall quantification of these bonds show lower absorption loss with 40% reduction in Si–H absorption bonds as compared to conventional SiH_4_-based USRN films reported in literature. Fabricated DCS-based USRN waveguides and ring resonators present propagation loss of ~ 2.2 dB/cm for a 1.2 μm wide waveguide and intrinsic quality factors $\sim 1.53\times {10}^{5}$ (~ 2.5× higher than that of SiH_4_-based USRN), indicating that the 40% reduction in Si–H absorption bonds play a part in improving the device performance. Hence a good material is of primary importance to device performance. This work explores the performance of USRN photonics devices using CMOS-compatible low temperature plasma-deposited DCS-based USRN material platform, a relatively unexplored chemistry for USRN material. With increasing interest and excellent demonstrations of optical signal processing on the USRN material platform which has high nonlinearity, the improved DCS-based USRN performance demonstrated in this paper could potentially bring promise to better performance in USRN photonics devices and a new pathway to further improve overall performance, tailored by a judicious choice of material chemistries.

## Methods

### DCS-based USRN films

DCS-based USRN films are plasma-deposited using an inductively coupled plasma chemical vapor deposition (ICP-CVD, Oxford Instruments PlasmaPro System100) tool on 8-inch wafers at a substrate temperature of ~ 300 °C. The substrate wafer used is Si with a layer of 10 μm thick SiO_2_ thermal oxide. USRN films are deposited on top of the SiO_2_ thermal oxide layer using DCS and N_2_ gas precursors. Prior to USRN film deposition, the chamber is coated with a layer of SiO_2_ to prevent flaking from the chamber during the deposition process. Chamber pre-conditioning is also done to condition the chamber to DCS and N_2_ environment before introducing the substrate for USRN deposition. Upon completion of USRN deposition, a CMP process is done on the surface of the USRN film to reduce the film’s surface roughness. The morphology of these DCS-based USRN films are characterized using high resolution TEM (FEI Tecnai X-TWIN) and tapping mode AFM (Veeco DI 3100). For cross-sectional TEM imaging, samples are prepared by sputtering the DCS-based USRN film with Au and Pt before FIB (FEI DA 3100) cut. Once cut, cross-sectional TEM imaging and FFT are conducted to observe the film structure of DCS-based USRN film. To measure the smoothness of the film, AFM is used to scan the film surface roughness rms of DCS-based USRN film over a scan area of 3 μm by 3 μm using tapping mode AFM.

XPS (PHI Quantera SXM) is used to analyse the chemical composition of DCS-based USRN films. The XPS source used is Al Kα with spot size of 200 μm × 200 μm. The optical properties of DCS-based USRN films are characterized using a variable-angle spectroscopic ellipsometer (VASE, Woollam). The film is scanned across the wavelength range from 400 to 1600 nm at 3 different incident angles of 65°, 70° and 75° respectively. The wavelength is set to increase at a step size of 10 nm over the wavelength range during the measurement. Cody–Lorentz optical model^50^ is then used to fit the measured data to extract the film’s refractive index, *n* and extinction coefficient, *k*. Using ATR technique in FTIR (Shimadzu AIM-9000) spectroscopy, the film’s absorption bonds are identified and quantified. As the USRN films are grown on 10 μm thermal SiO_2_ on Si substrate, ATR technique can help to penetrate only into the USRN films due to its nature for thin films and surface layers characterization.

### Device design and fabrication for DCS-based USRN waveguide devices

Using the deposited DCS-based USRN films, we fabricate waveguide devices to quantify the propagation losses and performance of microresonators. The devices are fabricated with electron-beam lithography and reactive ion etching to transfer the patterned resist onto the films. Plasma enhanced chemical vapor deposition is then used for SiO_2_ cladding. Ring resonators with radius of 100 µm, various bus waveguide widths of 1000 nm and 1200 nm, and coupling gaps of 200 nm are fabricated in a 400 nm thick DCS-based USRN film. The racetrack length is 30 µm for the resonator with width of 1000 nm and 60 µm for the resonator with width of 1200 nm.
